# mSphere of Influence: How the single cell contributes to the collective

**DOI:** 10.1128/msphere.00431-24

**Published:** 2024-12-11

**Authors:** Gina R. Lewin

**Affiliations:** 1 Department of Pathology, Center for Global Health and Diseases, Case Western Reserve University School of Medicine, Cleveland, Ohio, USA; 2 Case Western Reserve University-Cleveland VA Medical Center for Antimicrobial Resistance and Epidemiology, Cleveland, Ohio, USA; University of Georgia, Athens, Georgia, USA

**Keywords:** single-cell RNA-seq, transcriptional heterogeneity, subpopulations

## Abstract

Gina Lewin works in the field of microbial ecology, with a focus on the human microbiota. In this mSphere of Influence article, she reflects on how two papers describing bacterial single-cell RNA-seq—“Prokaryotic single-cell RNA sequencing by *in situ* combinatorial indexing” by S. B. Blattman, W. Jiang, P. Oikonomou, and S. Tavazoie (Nat Microbiol 5:1192–1201, 2020, https://doi.org/10.1038/s41564-020-0729-6) and “Microbial single-cell RNA sequencing by split-pool barcoding” by A. Kuchina, L. M. Brettner, L. Paleologu, C. M. Roco, et al. (Science 371:eaba5257, 2021, https://doi.org/10.1126/science.aba5257)—impacted her work by developing a new approach to study how single cells of bacteria contribute to ecosystem-level processes.

## COMMENTARY

In microbiology, we know that size matters. Your typical bacterium is only ~1 µm^3^, and thus, the micron scale is the fundamental level at which bacteria interact with their environments, a level that is hard to appreciate as humans (1). This challenge is one of the things that draws me to microbiology. I love the puzzle of understanding how things work that are too small for us to see. Through much of my PhD and postdoctoral training, I used sequencing approaches, including RNA-seq, to show how bacteria behave across different environments. But I always wondered if I had captured the whole story. For example, when I analyzed the gene expression of an oral pathogen from tooth scrapings taken from people with periodontitis (2), the samples were prepared from millions of microbes across millimeters of a tooth surface. I would ask, are all cells of this pathogen producing the same virulence factors and eating the same carbon sources? Likely not. Not all bacteria are in an identical environment, surrounded by the same host and bacterial cells at the micron level. Furthermore, targeted studies have shown repeatedly that bacterial gene expression varies across clonal cells for traits such as growth rate, antibiotic resistance, and competence due to micron-scale environmental differences, but also bistability, stochasticity, and genealogical effects (3, 4). Then, in 2020 and 2021, two papers, by Blattman et al. (5,6) and Kuchina et al. (6,6), opened up a new way to study bacterial gene expression at this single-cell level using bacterial single-cell RNA-seq (scRNA-seq).

In their publications, the authors sequenced the gene expression within individual bacterial cells. scRNA-seq was popular with eukaryotic systems at the time, but bacteria presented unique challenges. Bacteria have recalcitrant cell walls and low numbers of mRNA per cell, and they lack polyadenylated mRNAs. The Blattman and Kuchina strategies overcame these issues in an untargeted and unbiased manner that does not require probes or primers for certain bacteria or specific genes of interest. Furthermore, their approaches are high-throughput and can sequence tens of thousands of cells at a time, an improvement on previous methods that required cell sorting using microfluidics. Briefly, the scRNA-seq methods first fix and permeabilize cells. Then, cells are split randomly across successive 96-well plates in a process called “split-pool barcoding” that tags each cell’s cDNA with a unique set of barcodes, so that cDNAs from the same cell all have the same barcode combination, and cDNAs from different cells all have different barcode combinations. After library preparation and sequencing, these unique barcodes allow the researcher to identify which transcripts came from the same cells. By applying these approaches to both Gram-positive and Gram-negative bacteria, the authors showed that gene expression is heterogeneous across diverse bacteria. For example, *Bacillus subtilis* was heterogeneous in its expression of genes related to motility, antimicrobial production, and carbon metabolism (6).

However, beyond what these papers found, their publication created the possibility to understand bacteria at a new level. Up to this point, we only have peeked into the transcriptional heterogeneity of bacteria. There is so much unknown, and so many aspects of bacterial biology that we can better understand by studying single cells instead of averaging across a whole population. These techniques now allow my lab to study the role of single cells in population- and community-level phenotypes, drastically influencing the questions we pursue. How does transcriptional heterogeneity impact antimicrobial responses? How does transcriptional heterogeneity influence microbe-host interactions? And a question my lab is particularly interested in, how does transcriptional heterogeneity affect interactions within microbial communities? There are also fundamental questions to address, including about (i) the factors that drive heterogeneity, (ii) when single-cell-level heterogeneity matters at a community or ecosystem level, and (iii) the relationships between protein heterogeneity and transcriptional heterogeneity. I am excited to dive into these questions and the many more that I am sure will develop over time.

In addition, there are still opportunities to learn how to improve scRNA-seq approaches and analyses. Since these initial papers, researchers have continued to refine scRNA-seq approaches in bacteria by using droplet-based 10× Genomics technologies, which allows for sequencing more cells, and by depleting rRNAs, which decreases sequencing costs (7–9). Furthermore, the initial scRNA-seq papers primarily applied standard eukaryotic scRNA-seq analysis pipelines to their bacterial data sets. While this was successful, more recent papers have expanded the ways that we can analyze bacterial scRNA-seq data. For example, by combining droplet-based scRNA-seq approaches and a community-focused computational pipeline, Jia et al. (10) were able to study functional heterogeneity across 174,531 microbial cells from 2,534 species directly from the rumen microbiome. Also, Pountain et al. (11) recently used scRNA-seq approaches to study the relationship between gene transcription and genome replication, showing the capability to apply scRNA-seq to ask diverse questions across microbiology. Finally, each scRNA-seq experiment generates new hypotheses that can be tested in the lab. I am optimistic that the study of bacterial gene expression at the level of the single cell, piloted by these two key initial papers, will dramatically expand our understanding of microbiology.
